# Design of a 3DOF XYZ Bi-Directional Motion Platform Based on Z-Shaped Flexure Hinges

**DOI:** 10.3390/mi13010021

**Published:** 2021-12-24

**Authors:** Jinqiang Gan, Jiarong Long, Ming-Feng Ge

**Affiliations:** School of Mechanical Engineering and Electronic Information, China University of Geosciences, Wuhan 430074, China; longjr@cug.edu.cn (J.L.); gemf@cug.edu.cn (M.-F.G.)

**Keywords:** compliant mechanism, Z-shaped flexure hinges, differential moving, bi-directional motion

## Abstract

This paper presents a design of a 3DOF XYZ bi-directional motion platform based on Z-shaped flexure hinges. In the presented platform, bridge-type mechanisms and Z-shaped flexure hinges are adopted to amplify its output displacement. Bi-direction motion along the X-axis and Y-axis follows the famous differential moving principle $\left(\mathrm{DMP}\right)$, and the bi-directional motion along the Z-axis is realized by using the reverse arrangement of the Z-shaped flexure hinges along the X-axis and Y-axis. Statics analysis of the proposed platform is carried out by the energy method, compliance matrix method, and force balance principle. Meanwhile, the Lagrange method is used to analyze the dynamics of the platform. A series of simulations are conducted to demonstrate the effectiveness of the proposed design. The simulation results show that the average displacements of the platform in the XYZ-axis are ±125.58 μm, ±126.37 μm and ±568.45 μm, respectively.

## 1. Introduction

Precision positioning platforms have been widely used in bioengineering [1,2,3], precision optics [4,5,6], atomic force microscopes [7,8], aerospace [9] and other engineering fields [10,11,12]. They are playing an increasingly significant role in micro-nano operations. Conventional mechanisms can usually be divided into rigid mechanisms and compliant mechanisms. With the continuous development of science and technology, rigid mechanisms can no longer meet the current needs of high precision and rapid response on specific occasions due to their limitations, such as friction and clearance [13]. On the contrary, the compliant mechanisms with the advantages of no friction, no clearance, and high precision have become a research focus in the field of micro-nano operations in the past few years [14,15]. In micro-nano operations, electromagnetic actuators, electrostatic actuators, electrothermal actuators, and piezoelectric ceramic actuators are usually used to drive the platforms [16]. As one kind of the most popular actuators, piezoelectric ceramic actuators are widely used in precision platforms for their merits of high resolution, fast response speed, and large driving force [17,18]. The traditional compliant precision positioning platforms can be divided into a single degree of freedom platforms [19] and multi-degree of freedom platforms [20,21]. With the development of the micro-nano field, it is difficult for the single degree of freedom platforms to meet the requirements of current precise operation. Therefore, the research of high-performance multi-degree of freedom precision positioning platforms has become the research focus in these years [22,23].

The XYZ-platform is an important type of precise positioning platform, which is required in some space operations [24,25]. The spatial XYZ-platforms have been widely researched in the past few decades. For example, Lv et al. [26] introduced an innovative design based on a three-dimensional (3D) motion device, which can produce precise and fast micro-displacement. Zhu et al. [24] adopted the orthogonal arrangement of a three-chain parallel mechanism to achieve the low coupling and high natural frequency of XYZ-axis. Xu et al. [27] also designed an XYZ precise positioning platform by using the orthogonal arrangement of three chains and introduced a multistage lever amplification mechanism in each chain to amplify the output displacement. Zhang et al. [28] amplified the stroke of XYZ platform for more than 30 times than the input displacement through the bridge lever compound amplification mechanism. It should be noted that most of these Spatial XYZ platforms consist of three orthogonal chains along the XYZ directions, which generally leads to their bigger overall sizes [29,30]. Furthermore, the bigger size will also cause other problems such as greater mass, which has a negative impact on the natural frequency of the platform.

To reduce the size of the platform, Ling et al. [31] placed a piezoelectric ceramic actuator and two-stage amplification mechanism in the center of the planar mechanism. Zhang et al. [20] adopted the zigzag beams and using the differential moving principle (DMP) to realize XYZ movement with compact structure size. Ghafarian et al. [32] designed a circular small-size XYZ precision positioning platform with three bridge-type mechanisms arranged in a 120-degree plane and inclined blocks with semicircular notch hinges. Wang et al. [33] design a near-plane structure, the high natural frequency XYZ platform adopted the wedge structure in each of the three chains. These platforms have been reduced the size in different ways. However, they can not achieve a large stroke in all three degrees of freedom simultaneously. Moreover, they can only achieve two degrees of freedom bi-directional motion at most.

The Z-shaped flexure hinge is originally proposed by Guan et al. [34], which is used as a driver. It can change the direction of motion in a compact structure and amplify the stroke in the output direction. Subsequently, some researchers have also designed various platforms using Z-shaped flexure hinges. Liu et al. [35] placed a piezoelectric ceramic actuator at the output end of the Z-shaped flexure hinges in a special structure to achieve the movement of the XYZ axis. Xie et al. [36] used three actuators and the symmetrical Z-shaped flexure hinges structure to achieve 3DOF movement in a planar mechanism. With the rapid development of micro-nano operations, it is more difficult for the traditional platform only moving in one-direction to meet some cases which require positive and negative directions relative to its origin. Therefore, bi-directional motion platforms have been designed by some researchers. Choi et al. [37] designed a XY bi-directional motion platform for micro-nano operations. Zhu et al. [38] designed a 2DOF platform for tool cutting by using Z-shaped flexure hinges; it can realize a bi-directional cutting function on the X axis. In this paper, by using the compact structure of Z-shaped flexure hinges and their functions of displacement amplification and direction change, 3DOF bi-directional motion is realized in a nearly plane structure, and the platform has a large stroke along the Z axis.

To sum up, the main problem existing in the current XYZ precision positioning platforms is the mismatch between the platform size and the stroke. In particular, they can not simultaneously realize a larger stroke while reducing the size along the Z axis. Furthermore, most of the platforms cannot generate positive and negative bi-directional movement relative to the origin. To solve these problems, in this paper, a 3DOF XYZ bi-directional motion platform based on Z-shaped flexure hinges is presented. In this platform, the reverse arrangement of Z-shaped flexure hinges on the X-axis and Y-axis is introduced to acquire a near-plane structure, small size, large stroke and bi-directional motion in every degrees of freedom.

The rest of the paper is organized as follows: Section 2 introduces the structure design and principle of the platform; in Section 3, Z-shaped flexure hinges are analyzed, and the static and dynamic analysis of the platform is carried out; Section 4 carries on the finite element simulation to the platform to verify the rationality of the theoretical analysis; finally, Section 5 is the summary of the whole paper.

## 2. Design of the XYZ Bi-Directional Motion Platform

**Figure 1:**
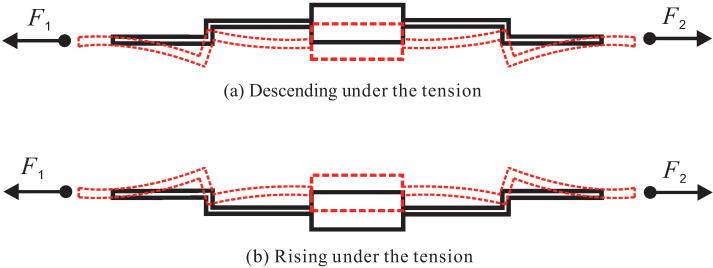
Z-shaped flexure hinge bending deformation principle.

**Figure 2:**
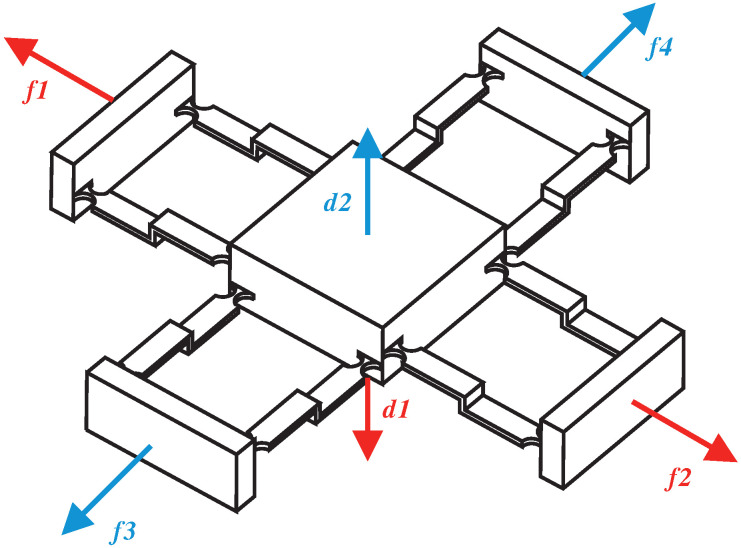
Z bi-directional movement by using Z-shaped flexure hinges.

In this paper, it is essential to rationally use and arrange the Z-shaped flexure hinges in order to realize XYZ bi-directional motion. The Z-shaped flexure hinges can change the direction of movement and amplify the output displacement by bending deformation. Figure 1 shows the working principle of Z-shaped flexure hinges. The rigid body is laid out in the middle, and symmetrical Z-shaped flexure hinges are arranged on both sides of the rigid body. Z-shaped flexure hinges would produce deformation because of bending when force or displacement to both ends is imposed. The direction of bending deformation is related to the layout of the Z-shaped flexure hinges. As shown in Figure 2, when a pair of Z-shaped flexure hinges with opposite directions are arranged on the X-axis and Y-axis, respectively, the moving platform in the middle can move up or down on the Z-axis relative to the origin.

**Figure 3:**
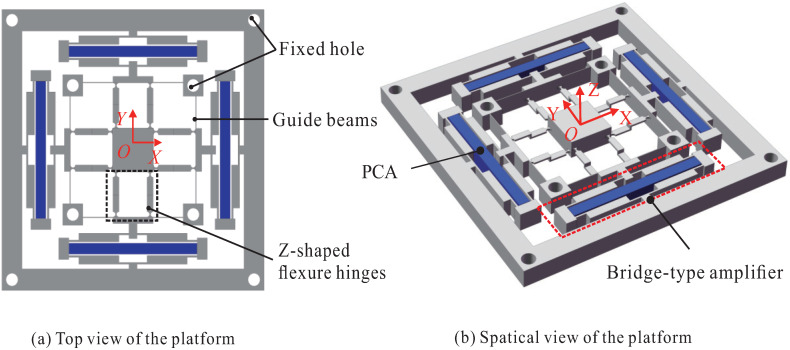
Structure design of the platform.

The structure design of the XYZ bi-directional motion platform is shown in Figure 3. The bridge-type amplification mechanisms in each of the four branched chains are used to enlarge the input displacements. The leaf-shaped flexure guide hinges are designed at the output end of the bridge mechanism to decouple the input and output. The Z-shaped flexure hinges are connected in series with the bridge-type mechanism and the leaf-shaped flexure guide hinges, and they are finally connected to the moving platform. The bridge-type mechanism, leaf-shaped flexure guide beams, and Z-shaped flexure hinges on each side form a branch chain, and the four chains form the precise positioning platform of the 4-PP configuration. It is worth noting that the four chains are not entirely symmetrical because the Z-shaped flexure hinges arranged in the X-axis and Y-axis are opposite.

In order to generate XYZ bi-direction movement, a pair of identical piezoelectric ceramic actuators (PCA) are arranged on the X-axis and Y-axis, respectively, as shown in Figure 3. According to the famous DMP, the difference between the driving forces of the two coaxial actuators will induce the motion of the platform along the X-axis or Y-axis [39]. The pair of actuators on the X-axis can motivate the platform to move in both positive and negative directions on the X-axis as well as a positive direction on the Z-axis. The pair of actuators on the Y-axis can motivate the platform to move in both positive and negative directions on the Y-axis as well as the negative direction on the Z-axis. In this way, it is feasible to achieve XYZ bi-directional motions with a nearly planar structure.

## 3. Modeling and Analysis of the Platform

The modeling and analysis of the 3DOF bi-directional motion platform are introduced in this section. The whole modeling is divided into two parts: statics and dynamics modeling. The statics modeling mainly includes the stiffness and amplification ratio analysis of the Z-shape flexure hinges, the stiffness analysis and the motion characteristics analysis of the whole platform. Dynamic modeling is mainly conducted to analyze the natural frequency of the platform.

### 3.1. Modeling of the Z-Shape Flexure Hinges

This section uses the compliance matrix method and energy method to analyze the compliance and amplification ratio of Z-shape flexure hinges. The compliance matrix method is a feasible methodology to establish the statics model of the compliant mechanism, and the details of the compliance matrix method can refer to Refs. [40,41].

#### 3.1.1. Compliance Model of the Z-Shape Flexure Hinges

**Figure 4:**
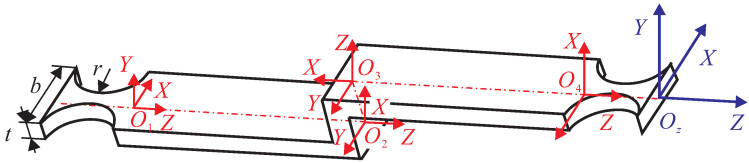
Schematic diagram of a Z-shaped flexure hinge.

As shown in Figure 4, the Z-shaped flexure hinge is composed of three leaf-type flexure hinges in series, and the middle one is perpendicular to the other two. In this paper, the semicircular notched hinges are adopted at the two ends of the Z-shaped flexure hinges to ensure their guiding function. Therefore, the Z-shaped flexure hinge can be regarded as a series of five hinges. The compliance of the five compliant hinges are added in series and converted to the $O_{5}$, which is the origin of the global coordinate system. The output compliance of the Z-shaped flexure hinge can be obtained as follows:

(1) $$C_{z}^{out}=C_{o_{1}}^{z}+C_{o_{2}}^{z}+C_{o_{3}}^{z}+C_{o_{4}}^{z}+C_{o_{5}}$$

where $C_{o_{i}}^{z}$(*i* = 1,2,3,4) denotes the compliance from the local coordinate system to the global coordinate system, which can be expressed as:

(2) $$C_{o_{i}}^{z}=Ad_{i}C_{o_{i}}A{d_{i}}^{T}$$

where $Ad_{i}$ denotes the adjoint matrix for coordinate transformation, which can be obtained by:

(3) $$Ad_{i}=\left[\begin{array}{cc} R & 0\\ \hat{t}R & R \end{array}\right]$$

where *R* denotes the rotation matrix from the local coordinate system to the global coordinate system, and $\hat{t}$ is the antisymmetric matrix defined by the translation vector:

(4) $$\hat{t}=\left[\begin{array}{ccc} 0 & -z & y\\ z & 0 & -x\\ -y & x & 0 \end{array}\right]$$

#### 3.1.2. Amplification Ratio of the Z-Shape Flexure Hinges

**Figure 5:**
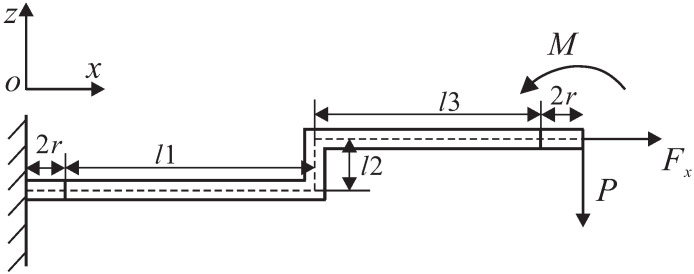
Single Z-shaped flexure hinge force diagram.

It is necessary to calculate the amplification ratio of Z-shaped flexure hinges because they have a decisive influence on the output displacement of the platform on the Z-axis. As shown in Figure 5, there are three reaction forces/moments, moment *M*, axial force $F_{x}$ and virtual force *P*. The matrix equation can be formulated according to the energy method, and the main equation is as follows:

(5) $$\left[\begin{array}{ccc} f11 & f12 & f13\\ f21 & f22 & f23\\ f31 & f32 & f33 \end{array}\right]\left[\begin{array}{c} F_{x}\\ P\\ M \end{array}\right]=\left[\begin{array}{c} \frac{\Delta x}{2}\\ \Delta z\\ 0 \end{array}\right]$$

where fij(i/j=1,2,3) denotes the compliance element associated with the all three of the hinges that form a Z-shaped flexure hinge. The expression of the output displacement on the Z-axis can be obtained from the compliance matrix:

(6) $$\Delta z=\frac{3\Delta x{\left(l_{1}+2r\right)}^{2}}{l_{2}^{2}+6\left(l_{1}+2r\right)l_{2}+\frac{2t^{2}\left(l_{1}+2r\right)}{l_{2}}}$$

According to the definition, the amplification ratio of the Z-shaped flexure hinge can be obtained from the following:

(7) $$A_{z}=\frac{\Delta z}{\Delta x}=\frac{3{\left(l_{1}+2r\right)}^{2}}{l_{2}^{2}+6\left(l_{1}+2r\right)l_{2}+\frac{2t^{2}\left(l_{1}+2r\right)}{l_{2}}}$$

The main parameters of the single Z-shaped flexure hinge are listed in Table 1, and the amplification ratio of Z-shaped flexure hinge can be obtained by substituting them into Equation (7).

### 3.2. Compliance Model of a 3DOF Bi-Directional Motion Platform

The compliance matrix method is used to analyze the output compliance and the input compliance in this section.

#### 3.2.1. Output Compliance of the Stage

The 3DOF bi-directional motion precision positioning platform is composed of four moving chains. Each chain comprises a bridge-type amplification mechanism, a pair of leaf-shaped flexure hinges, and a pair of Z-shaped flexure hinges. In addition, the positioning platform is symmetry in the coaxial. Thus, it just needs to calculate the output stiffness of one branch in both the X-axis and Y-axis.

**Figure 6:**
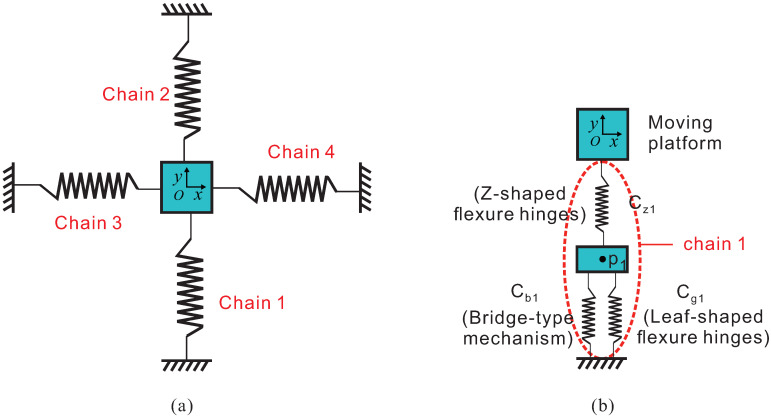
Output compliance model of the platform: (**a**) the total equivalent stiffness model; (**b**) the stiffness model contained in branch chain 1.

As shown in Figure 6, the whole platform consists of four chains which are marked by 1, 2, 3 and 4. Each of the chains has a similar construction and they are in parallel. Figure 6b shows that chain 1 is composed of the bridge-type mechanism, the leaf-shaped flexure hinges and the Z-shaped flexure hinges. The bridge-type mechanism is firstly connected in parallel with the leaf-shaped flexure hinges. Subsequently, they are connected in series with the Z-shaped flexure hinges. Finally, the Z-shaped flexure hinges are connected with the moving platform. In this section, the output compliance of chain 1 is firstly calculated. According to the parallel relationship, the compliance of the bridge-type mechanism and leaf-shaped flexure guide beams at point P1 can be obtained:

(8) $$C_{P1}={\left[{\left(C_{b1}^{P1}\right)}^{-1}+{\left(C_{g1}^{P1}\right)}^{-1}\right]}^{-1}$$

where $C_{b1}^{P1}$ denotes the output compliance of the bridge-type mechanism at point P1, and $C_{g1}^{P1}$ denotes the output compliance of the leaf-shaped flexure guide beams at point P1. They can be expressed by:

(9) $$C_{b1}^{P1}={\left\{{\left(\sum_{i=1}^{4}C_{boi}^{P1}\right)}^{-1}+{\left[R_{y}\left(\pi \right)\left(\sum_{i=1}^{4}C_{boi}^{P1}\right)R_{y}{\left(\pi \right)}^{T}\right]}^{-1}\right\}}^{-1}$$

(10) $$C_{g1}^{P1}={\left({C_{gl}^{P1}}^{-1}+{C_{gr}^{P1}}^{-1}\right)}^{-1}$$

where $C_{boi}^{P1}$ denotes the output compliance of compliant hinge in bridge mechanism at point P1, $R_{y}\left(\pi \right)$ denotes the rotation change matrix $180^{\circ }$ along the Y-axis, ${C_{gl}^{P1}}^{-1}$ and ${C_{gr}^{P1}}^{-1}$ denote the output compliance of the left and right leaf-shaped beams of the guide mechanism, respectively. Using the compliance matrix method to calculate the output stiffness of Z-shaped flexure hinge at a certain point has been introduced in Section 3.1.1. The compliance of the Z-shaped flexure hinges at point P1 can be expressed as:

(11) $$C_{z}^{P1}={\left\{{\left(\sum_{i=1}^{5}Ad_{i}C_{oi}^{Z1}Ad_{i}^{T}\right)}^{-1}+{\left(\sum_{i=1}^{5}Ad_{i}C_{oi}^{Z2}Ad_{i}^{T}\right)}^{-1}\right\}}^{-1}$$

where $C_{oi}^{Z1}$ and $C_{oi}^{Z2}$ denote the compliance of the Z-shaped flexure hinges in the local coordinate system of chain 1. Therefore, the compliance of chain1 at point P1 can be obtained as follows:

(12) $$C_{P1}^{out}=C_{P1}+C_{z}^{P1}$$

The compliance of chain 1 at point *P* is converted to the center point *O* of the moving platform:

(13) $$C_{P1}^{o}=Ad_{P1}C_{p}^{out}Ad_{P1}^{T}$$

Since branch chain 2 at coaxial is symmetric with branch chain 1, the compliance of branch chain 2 at point *O* can be obtained by using a rotation transformation matrix as follows:

(14) $$C_{P2}^{o}=R_{z}\left(\pi \right)C_{P1}^{o}R_{z}{\left(\pi \right)}^{T}$$

Thus, the compliance of the platform along the Y axis can be expressed by:

(15) $$C_{o}^{y}={\left[{\left(C_{P1}^{o}\right)}^{-1}+{\left(C_{P2}^{o}\right)}^{-1}\right]}^{-1}$$

By using the same method, the compliance of branch chains 3 and 4 on the X-axis is converted to the *O* point in the center of the terminal moving platform. Then, the compliance of all of the branch chains on the X-axis and Y-axis can be superimposed. Finally, the output stiffness of the moving platform can be obtained as:

(16) $$C_{o}={\left[{\left(C_{o}^{x}\right)}^{-1}+{\left(C_{o}^{y}\right)}^{-1}\right]}^{-1}$$

where $C_{o}^{x}$ and $C_{o}^{y}$ denote the compliance of the mechanism along the X axis and the Y axis at the center point *O* of the moving platform, respectively.

#### 3.2.2. Input Compliance of the Stage

**Figure 7:**
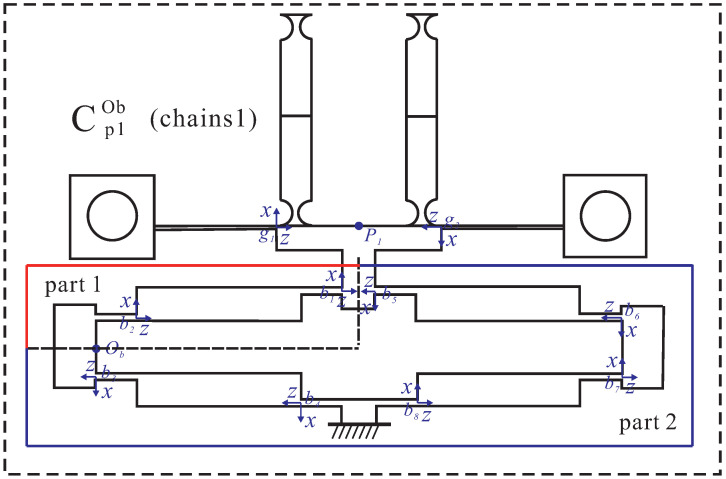
Input compliance of the stage.

Since the compliance of the four branch chains of the platform are nearly equal, the input compliance of the platform at the bridge-type mechanism in branch chain 1 on the Y-axis can be calculated as an example. As shown in Figure 7, branch chains 2, 3, and 4 can be regarded as parallel chains and they are connected in series to branch chain 1. Firstly, the compliance of the three parallel chains 2, 3, and 4 at $O_{b}$ point can be calculated as:

(17) $$C_{ex-p_{1}}^{o_{b}}={\left[{\left(Ad_{x}C_{o}^{x}Ad_{x}^{T}\right)}^{-1}+{\left(Ad_{p}C_{p2}^{o}Ad_{p}^{T}\right)}^{-1}\right]}^{-1}$$

where $Ad_{x}$ denotes the transformation matrix of the compliance from the center point *O* of the platform to the input point $O_{b}$ of branch chains 3 and 4 on the X-axis. $Ad_{p}$ is the transformation matrix of the compliance from the center point *O* of the platform to the input point $O_{b}$ of branch chain 2 on the Y-axis. In addition, then the compliance of Z-shaped flexure hinges, guide mechanisms, and bridge-type mechanism compliant hinges at point $O_{b}$ in branch chain 1 can be calculated by:

(18) $$C_{p}^{o_{b}}={\left\{{\left\{{\left[{\left(C_{z}^{o_{b}}\right)}^{-1}+{\left(C_{g}^{o_{b}}\right)}^{-1}+{\left(\sum_{i=5}^{8}C_{b_{i}}^{o_{b}}\right)}^{-1}\right]}^{-1}+\sum_{i=1}^{2}C_{b_{i}}^{o_{b}}\right\}}^{-1}+{\left(\sum_{i=3}^{4}C_{b_{i}}^{o_{b}}\right)}^{-1}\right\}}^{-1}$$

where $C_{z}^{o_{b}}$ and $C_{g}^{o_{b}}$ denote the compliance of Z-shaped flexure hinges from the local coordinate system to point $O_{b}$ and the compliance of the leaf-shaped flexure beams from local coordinate system to point $O_{b}$, respectively. $C_{b_{i}}^{o_{b}}$ denotes the compliance of the hinges in a bridge-type mechanism from a local coordinate system to point $O_{b}$. Thus, the input compliance of the platform at point $O_{b}$ can be obtained by:

(19) $$C_{in}=C_{ex-p}^{o_{b}}+C_{p}^{o_{b}}$$

### 3.3. Analysis of Motion Characteristics of the XYZ Bi-Directional Motion Platform

This section mainly discusses the relationship between the input displacement of the platform and the output displacement of each mechanism of the platform. Since the motion characteristic of the platform on the X-axis and Y-axis is almost equivalent, only the motion characteristics in the X-axis as an example in this section are analyzed.

**Figure 8:**
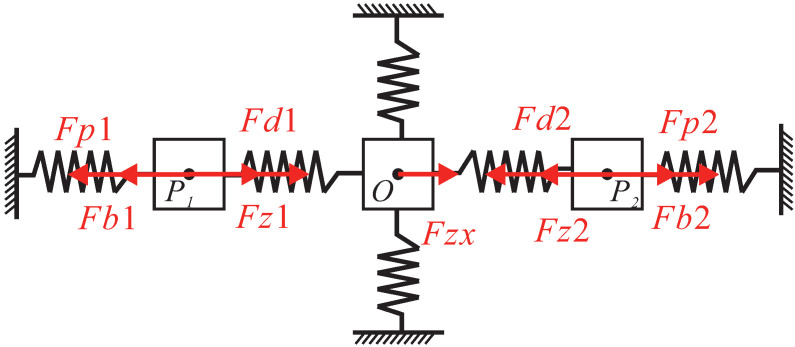
Equivalent rigid body model on the X-axis.

The equivalent rigid body and force diagram of motion characteristics on the X-axis of the platform is shown in Figure 8. The flexure hinges are regarded as springs on the X-axis, and the Z-shaped flexure hinges on Y-axis are also regarded as springs. In this case, one of the actuators or both of them can be driven on the X-axis.

Firstly, the case of driving only the left actuator would be discussed in this section. The following equation can be obtained according to the principle of energy conservation:

(20) $$\begin{array}{c} \frac{1}{2}F_{p1}X_{bl}^{out}=\frac{1}{2}K_{g}{X_{bl}^{out}}^{2}+\frac{1}{2}K_{z}{\left(X_{bl}^{out}-X_{out}\right)}^{2}+\frac{1}{2}K_{z}^{x}X_{out}^{2}\\ +\frac{1}{2}K_{g}X_{r}^{2}+\frac{1}{2}K_{b}X_{r}^{2}+\frac{1}{2}K_{z}{\left(X_{out}-X_{r}\right)}^{2}+\frac{1}{2}K_{z}^{y}Z_{out}^{2} \end{array}$$

where $F_{p1}$ denotes equivalent driving force at the output of bridge-type mechanism. $X_{bl}^{out}$ denotes the output displacement of the bridge mechanism on the left, and $K_{g}$ denotes the stiffness of leaf-shaped guide beams. $K_{z}$ denotes the stiffness of the Z-shaped flexure hinges along the X-axis, and $K_{z}^{x}$ denotes the stiffness of Z-shaped flexure hinges on the Y-axis in their moving direction. $X_{out}$ denotes the displacement of the moving platform in the X direction. $X_{r}$ denotes the displacement of the bridge-type mechanism on the right. $K_{b}$ denotes the stiffness of the bridge-type mechanism, $K_{z}^{y}$ and $Z_{out}$ denote the stiffness on the Z-axis of the platform and the output displacement on the Z-axis of the moving platform, respectively. The stiffness of the mechanism mentioned above can be calculated by using the compliance matrix method.

**Figure 9:**
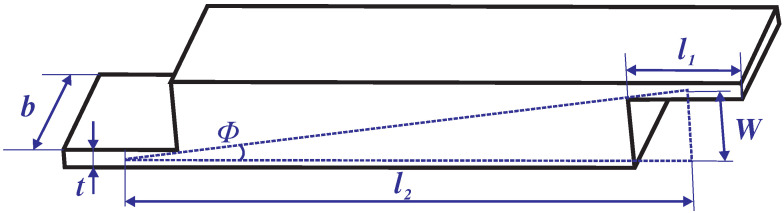
The bridge-type mechanism.

In addition, to obtain the parameters $F_{p1}$ and $X_{bl}^{out}$, the bridge-type mechanism as shown in Figure 9 needs to be analyzed separately. Firstly, the amplification ratio of the bridge mechanism is calculated. Xu et al. [42] modeled the amplification ratio of the bridge-type mechanism accurately by considering the stiffness of compliant hinge. This modeling method is adopted in this paper to calculate the amplification ratio of the bridge-type mechanism:

(21) $$A_{b}=\frac{K_{t}l_{2}^{2}cos^{3}\Phi sin\Phi }{2K_{r}+K_{t}l_{2}^{2}cos^{2}\Phi sin^{2}\Phi }$$

where $K_{t}$ and $K_{r}$ denote the translational and rotational stiffnesses of the flexure hinges, respectively. $l_{2}$ denotes the straight-line distance between two compliant hinges of a quarter bridge-type mechanism, and Φ denotes the angle between the line between two compliant hinges and the horizontal line. The main parameters of the bridge mechanism are shown in Table 2. Thus, $F_{p1}$ can be expressed as:

(22) $$F_{p1}=X_{b}K_{b}=A_{b}X_{in}K_{b}$$

where $X_{in}$ denotes the input displacement of the bridge-type mechanism. When the bridge mechanism is connected to the external structure, its output displacement can be obtained by:

(23) $$X_{bl}^{out}=\frac{X_{b}K_{b}}{K_{b}+K_{exb}}=\frac{A_{b}X_{in}K_{b}}{K_{b}+K_{exb}}$$

where $K_{exb}$ denotes the stiffness of all other mechanisms of the platform at point P1 except the bridge mechanism on the left. In addition, the output displacement of the moving platform along the Z direction can also be obtained as follows:

(24) $$Z_{out}=A_{z}\left(X_{bl}^{out}-X_{r}\right)$$

According to the force balance principle on the X direction, the following equation can be obtained:

(25) $$\begin{array}{cc} F_{b}^{out} & =F_{gl}+F_{zl}+F_{z}^{x}+F_{gr}+F_{br}+F_{zr}\\ \; & =K_{g}X_{bl}^{out}+K_{z}\left(X_{bl}^{out}-X_{out}\right)+K_{z}^{x}X_{out}+K_{g}X_{r}+K_{b}X_{r}+K_{z}\left(X_{out}-X_{r}\right) \end{array}$$

where $F_{gl}$ denotes the reaction force of the left leaf-shaped guide beams, $F_{zl}$ denotes the reaction force of the left Z-shaped flexure hinges and $F_{z}^{x}$ denotes the reaction force of the Z-shaped flexure hinges on the Y-axis. $F_{gr}$ denotes the reaction force of the right leaf-shaped guide beams, Fbr denotes the reaction force of the right bridge mechanism, and $F_{zr}$ denotes the reaction force of the right Z-shaped flexure hinges. Combining Equations (20) and (25), $X_{out}$ and $X_{r}$ can be solved:

(26) $$\left\{\begin{array}{c} X_{out}=6.47X_{inl}\\ X_{r}=6.17X_{inl} \end{array}\right.$$

Secondly, the case of driving both of the actuators on the X-axis would be discussed. In this case, equivalent driving forces exist at the output of the bridge-type mechanism on both sides along the X-axis. The force balance relationship is as follows:

(27) $$F_{pl}-F_{pr}=\left(F_{b}^{r}+F_{g}^{l}+F_{z}^{l}+F_{z}^{x}\right)-\left(F_{b}^{l}+F_{g}^{r}+F_{z}^{r}\right)$$

where $F_{pl}$ and $F_{pr}$ denote the equivalent driving force at the output of the bridge-type mechanism on both sides of the actuator, respectively. They can be expressed by:

(28) $$\left\{\begin{array}{c} F_{pl}=K_{b}A_{b}X_{inl}^{ideal}\\ F_{pr}=K_{b}A_{b}X_{inr}^{ideal} \end{array}\right.$$

where $A_{b}$ denotes the amplification ratio of the single bridge-type mechanism, and $X_{inl}^{ideal}$ and $X_{inr}^{ideal}$ denote the ideal input displacement of the single bridge-type mechanism. When connected with other mechanisms and external force is imposed, the input displacement of bridge-type mechanism is real input displacement $X_{in}^{real}$. The relation between the real input displacement $X_{in}^{real}$ and the ideal input displacement $X_{in}^{ideal}$ can be expressed by:

(29) $$X_{in}^{real}=X_{in}^{ideal}-X^{\prime }$$

where $X^{\prime }$ denotes the input end displacement of the bridge-type mechanism when it is imposed external force. Therefore, Equation (27) can be further written as:

(30) $$\begin{array}{c} K_{b}A_{b}\left(X_{inl}^{real}+X_{r}^{\prime }-X_{inr}^{real}-X_{l}^{\prime }\right)=[K_{b}X_{br}^{out^{\prime }}+K_{g}X_{bl}^{out^{\prime }}+K_{z}\left(X_{bl}^{out^{\prime }}-X_{out^{\prime }}\right)\\ +K_{z}^{x}X_{out}^{\prime }]-\left[K_{b}X_{bl}^{out^{\prime }}+K_{g}X_{br}^{out^{\prime }}+K_{z}\left(X_{br}^{out^{\prime }}-X_{out^{\prime }}\right)\right] \end{array}$$

where $X_{bl}^{out^{\prime }}$ and $X_{br}^{out^{\prime }}$ represent the displacement of the output end of the left and right bridge-type mechanism on the X-axis, respectively. $X_{out^{\prime }}$ represents the output displacement of the moving platform along the X direction. $X_{bl}^{out^{\prime }}$ and $X_{br}^{out^{\prime }}$ can be obtained by:

(31) $$\left\{\begin{array}{c} X_{bl}^{out^{\prime }}=X_{bl}^{out}-X_{b}l\left(inr\right)\\ X_{br}^{out^{\prime }}=X_{br}^{out}-X_{b}r\left(inl\right) \end{array}\right.$$

where $X_{b}l\left(inr\right)$ and $X_{b}r\left(inl\right)$ denote the output end displacement of the bridge-type mechanism when external force is imposed. The relationship between the displacement of moving platform along the X direction and the real input placement on both sides $X_{inl}^{real}$ and $X_{inr}^{real}$ can be obtained:

(32) $$X_{out^{\prime }}=3.03\left(X_{inl}^{real}-X_{inr}^{real}\right)$$

Meanwhile, the output displacement along the Z-axis can also be calculated from the output displacement of the bridge-type mechanism on both sides:

(33) $$Z_{out^{\prime }}=A_{z}\left(X_{bl}^{out^{\prime }}+X_{br}^{out^{\prime }}\right)$$

### 3.4. Dynamics Analysis of the 3DOF Bi-Directional Stage

The natural frequency of the platform would be analyzed by the concentrated mass method. X, Y, and Z at the output end of the moving platform are defined as the generalized coordinates u[x,y,z]. The Lagrange equation of the system can be expressed as:

(34) $$\frac{d}{dt}\left(\frac{\partial T}{\partial \dot{u}}-\frac{\partial T}{\partial u}+\frac{\partial V}{\partial u}\right)=F$$

*T* as the total kinetic energy of the system can be obtained by:

(35) $$T=\frac{1}{2}\left(M_{x}{\dot{x}}^{2}+M_{y}{\dot{y}}^{2}+M_{z}{\dot{z}}^{2}\right)$$

where $M_{x}$, $M_{y}$ and $M_{z}$ denote the equivalent mass along the X, Y, Z directions, respectively. Since the X and Y directions are nearly symmetric, it can be considered that $M_{x}$=$M_{y}$. In addition, the output displacement x is determined by the input displacement $x_{inl}$ and $x_{inr}$ along the X-axis.The output displacement y is determined by the input displacement $y_{inu}$ and $y_{ind}$ along the Y axis. The output displacement z is determined by all of the four input displacements. The potential energy of the system can be obtained by:

(36) $$V=\frac{1}{2}K_{xl}x_{inl}^{2}+\frac{1}{2}K_{xr}x_{inl}^{2}+\frac{1}{2}K_{yu}y_{inu}^{2}+\frac{1}{2}K_{yd}y_{ind}^{2}$$

where $K_{xl}$ and $K_{xr}$ denote the input stiffness of the bridge-type mechanism along the X-axis; $K_{yu}$ and $K_{yd}$ denote the input stiffness of bridge-type mechanism along the Y-axis. The undamped vibration equation of the mechanism is:

(37) $$M_{i}\dot{u}+K_{i}u=F\left(i=x,y,z\right)$$

The natural frequency equation is further derived:

(38) $$f=\frac{1}{2\pi }\sqrt{\frac{K_{i}}{M_{i}}}\left(i=x,y,z\right)$$

## 4. Finite Element Analysis and Discussion

**Figure 10:**
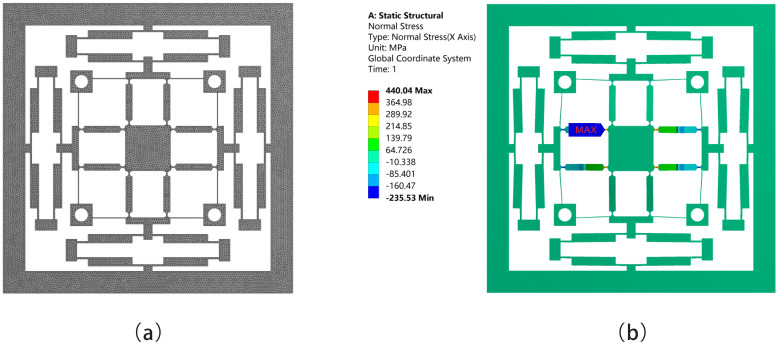
Meshing method and maximum stress: (**a**) meshing method of the platform; (**b**) the maximum stress.

The finite element analysis (FEA) is carried out by using ANSYS to test the performance of the proposed platform. In FEA, Al7075-T6 is chosen as the material of the platform, and its main properties are shown in Table 3. The reasons for use Al 7075-T6 are due to its low density and larger $\sigma_{s}/E$ ratio value, which make it have the merit of light weight and higher elasticity. In addition, $\sigma_{s}$ denotes the yield stress, *E* denotes the Young’s modulus. In addition, the platform adopts the automatic meshing method in ANSYS, and the size of the mesh is 0.5 mm. Figure 10a shows the meshing of the platform. In addition, Figure 10b shows the maximum stress of 440 MPa and it is lower than 505 MPa, which is the yield stress of Al 7075-T6. The maximum stress occurs when input displacement of 40 μm is applied to each side of the bridge-type mechanisms on the X direction. It appears on the semicircular notched hinges which connect the Z-shaped flexure hinges and the moving platform.

**Figure 11:**
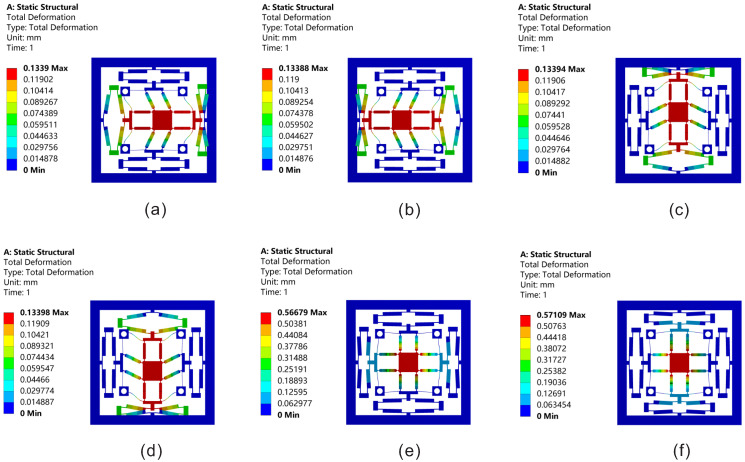
Total displacements along the XYZ three directions: (**a**) along X-axis positive direction; (**b**) along X-axis negative direction; (**c**) along Y-axis positive direction; (**d**) along Y-axis negative direction; (**e**) along Z-axis positive direction; (**f**) along Z-axis negative direction.

Firstly, the statics simulation of the platform is carried out in this section. Figure 11 shows the total deformation along the XYZ axis. If the platform needs to move along the X or Y axis, input displacement is supposed to be applied on one of the bridge-type mechanisms along the X or Y axis; meanwhile, the maximum input displacement is 20 μm. As shown in Figure 11a–d, an input displacement of 20 μm is applied to only one of the bridge-type mechanisms along the X axis or Y axis. In addition, the probe function is used in ANSYS to detect the displacements of each degree of freedom. The maximum displacements of the moving platform are 125.34 μm and −125.81 μm in positive and negative directions along the X-axis, respectively. The maximum displacements are 126.19 μm and −126.54 μm in positive and negative directions along the Y-axis. As shown in Figure 11e,f, if the platform needs to move along the Z-axis, input displacements should be applied on both sides of the bridge-type mechanisms on the X or Y axis. Meanwhile, both sides of the the maximum input displacements are 40 μm. The maximum positive and negative displacements on the Z-axis are 566.03 μm and −570.86 μm, respectively.

**Figure 12:**
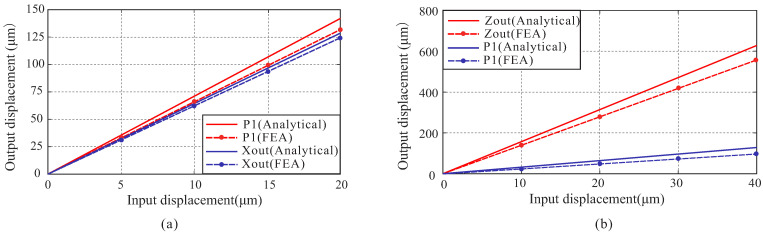
Relationship between the input displacement and output displacement: (**a**) only the left side of the X-axis applies input displacement; (**b**) both sides of X-axis have input displacements applied.

**Figure 13:**
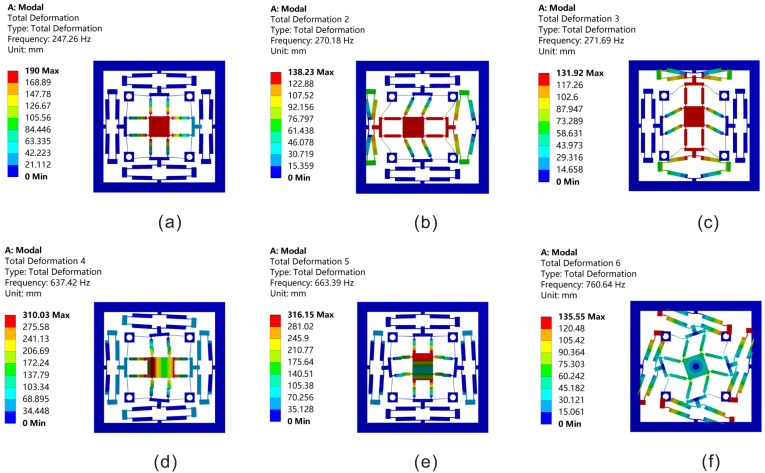
The first six natural frequencies of the platform: (**a**) the first natural frequency; (**b**) the second natural frequency; (**c**) the third natural frequency; (**d**) the fourth natural frequency; (**e**) the fifth natural frequency; (**f**) the sixth natural frequency.

Figure 12 shows the relation between the input displacement and output displacement, which is calculated by theoretical analysis and FEA, respectively. P1 denotes the output displacement of the left bridge-type mechanism, Xout denotes the output displacement of the moving platform along the X-axis, and Zout denotes the output displacement of the moving platform along the Z-axis. It can be seen from Figure 12b that the amplification of the bridge-type mechanism is less than the case of Figure 12a. It is because the bridge-type mechanism is subjected to bigger external force when both of the actuators along the X-axis are driven, which will reduce their displacements. The first six modes of the platform are simulated by using ANSYS. As shown in Figure 13, the first three natural frequencies are 247.3 Hz, 270.2 Hz and 271.7 Hz, respectively, which can meet the demands of this platform.

The input stiffness and output stiffness are simulated by FEA and the comparisons between theoretical analysis and simulation results are shown in Table 4. The error of the input stiffness between the FEA and analytical model is only 8.07%. The error of output stiffness on the X-axis, Y-axis and Z-axis are 5.67%, 3.73% and 12.74 %. These errors are small enough to show that the theoretical analysis is right. The error of the output stiffness on the Z-axis is the largest compared to that on the X-axis and the Y-axis. It is mainly because the compliance matrix method does not take the large deformation of the hinges into consideration and the displacement on the Z-axis is larger than that on the X-axis and Y-axis. In total, the simulations show that the proposed 3DOF XYZ bi-directional platform is effective.

Table 5 shows the comparisons between the developed platform and several typical XYZ-platforms at a similar size. It can be found that the size on the Z-axis of this platform is the smallest (except for Xie [36]) at a similar size on X and Y axes. In addition, this platform can provide a large stroke on the Z-axis of 1 mm (including both the positive and negative directions). In addition, the platform in this paper can generate positive and negative bi-directional motion relative to the origin at each degree of freedom of XYZ.

## 5. Conclusions

A new 3DOF XYZ precision positioning platform based on Z-shaped flexure hinges is firstly presented in this paper. The platform can achieve bi-directional movement on the X, Y, and Z-axis relative to the origin using four piezoelectric ceramic actuators. Bridge-type mechanisms and Z-shaped flexure hinges are adopted to acquire a large stroke especially on the Z-axis of the platform. Subsequently, the static analysis of the platform is carried out by using energy method, compliance matrix, force balance principle and energy conservation. Finally, simulations by FEA are carried out to verify the analytical models and the developed stage. The errors of both the input stiffness and output stiffness are less than 12.74%. The simulation results show that the average stroke of the platform along the X, Y, and Z-axis are ±125.58 μm, ±126.37 μm, ±568.45 μm, respectively. Its first three natural frequencies are 247.3 Hz, 270.2 Hz, 271.7 Hz, respectively. In a word, the developed platform in this paper has the merits of compact structure, large stroke and bi-directional motion on all the X, Y, and Z-axes. However, the proposed platform also faces some challenges such as being relatively difficult to manufacture, having a high cost and being difficult to control for four actuators. In the future, we will optimize the design of the Z-shaped flexure hinge and process a platform prototype for experiments.

Table 6 shows a nomenclature table for some parameters.

## Figures and Tables

**Table 1:** Main parameter of the Z-shaped flexure hinge.

Parameter	$l_{1}$ (mm)	$l_{2}$ (mm)	$l_{3}$ (mm)	*t* (mm)	*b* (mm)	*r* (mm)
Value	8	1.8	8	0.7	3	1.25

**Table 2:** Main parameters of the bridge-type mechanism.

Parameter	$l_{1}$ (mm)	$l_{2}$ (mm)	$l_{3}$ (mm)	*w* (mm)	*t* (mm)
Value	5	25	2.8	8	0.8

**Table 3:** Properties of Al7075-T6.

Parameter	Yield Strength (MPa)	Poisson’s Ratio	Density (kg/m${}^{3}$)	Young’s Modulus (MPa)
Value	505	0.33	2.81 × 10^3^	7.1 × 10^4^

**Table 4:** Comparisons of FEA and the Analytical model.

Method	Input Stiffness (N/µm)	Output Stiffness (N/mm)
Analytical model	8.17	X:76.24; Y:73.19; Z:27.78
FEA	7.56	X:72.15; Y:70.56; Z:31.32
Error	8.07%	X:5.67%; Y:3.73%; Z:12.74%

**Table 5:** Comparisons of 3DOF XYZ Platforms.

Platform	Input (µm)	Work Space (µm)	Size (mm)	Bi-Direction
Tang [29]	36/36/36	10.39/15.43/15.55	228/158/84	no
Zhang [20]	12.5/12.5/12.5/12.5	±72.8/±72.8/113.6	132.9/132.9/10	X and Y
Xie [36]	60/60/60	56/±29.7/265.62	145/145/6	Y
Tian [43]	15/15/15	128.1/131.3/17.9	134/134/27	no
This paper	40/40/40/40	±125.58/±126.37/±568.45	130.6/130.6/9	X, Y and Z

**Table 6:** Nomenclature table.

Notation	Meanig
$Ad_{i}$	The adjoint matrix for coordinate transformation
$\hat{t}$	The antisymmetric matrix
$A_{z}$	Amplification ratio of Z-shaped flexure hinges
$C_{in}$	Input compliance of the platform
$C_{o}$	Output compliance of the platform
$A_{b}$	Amplification ratio of the single bridge-type mechanism
*f*	Natural frequency of the platform
$\sigma_{s}$	Yield stress of Al7075-T6
*E*	Young’s modulus of Al7075-T6

## Data Availability

Not applicable.
